# Videoconference-Based Adapted Physical Exercise Training Is a Good and Safe Option for Seniors

**DOI:** 10.3390/ijerph18189439

**Published:** 2021-09-07

**Authors:** Olga Kuldavletova, Florane Pasquier, Lucile Bigot, Antoine Langeard, Antoine Gauthier, Gaëlle Quarck

**Affiliations:** 1 Health Department, Université de Caen Normandie, INSERM, COMETE, GIP Cyceron, 14000 Caen, France; olga.kuldavletova@unicaen.fr (O.K.); florane.pasquier@unicaen.fr (F.P.); lucile.bigot@mooven.fr (L.B.); antoine.langeard@unicaen.fr (A.L.); antoine.gauthier@unicaen.fr (A.G.); 2 Physical Activity and Sports Department, Université de Caen Normandie, INSERM, COMETE, GIP Cyceron, 14000 Caen, France

**Keywords:** videoconference, exercise, functional capacities, postural control, sensory information, seniors, aging

## Abstract

Videoconference-based adapted physical exercise combines the benefits of supervised exercise training with staying at home, when conventional training is inaccessible. However, exercising with the use of a screen can be considered an optokinetic stimulation, and could therefore induce changes in sensory processing, affecting postural stability. The objectives of this study were to compare the effectiveness of the training delivered Face-to-Face and by Videoconferencing in improving physical capacities of older adults, and to evaluate the possible effects of the Videoconference mode on the processing of sensory information that could affect postural control. Twenty eight older adults underwent the supervised exercise program for sixteen weeks either Face-to-Face or by Videoconference. Muscular strength of knee and ankle flexors and extensors, maximum oxygen uptake, postural stability and horizontal rotational vestibulo-ocular reflex were evaluated before and after the training. Both modes of training similarly increased the VO_2_ peak and strength of the motor muscles of lower limbs in all participants. The use of the Videoconference did not modify the vestibulo-ocular reflex in subjects or the importance of vision for postural control. Therefore, the Videoconference-based exercise training can be considered a safe and effective way to maintain good functional capacity in seniors.

## 1. Introduction

One-third of people aged 65 years old and older fall at least once a year [1,2]. From the age of 80, this concerns up to 50% [3,4]. The impact of falls can be dramatic for older adults, leading to hospitalization, loss of independence, or even lethal outcome. The reasons are numerous: advanced age is often accompanied by a decrease in functional capacities characterized by a reduction of strength, agility, flexibility and maximal oxygen uptake, as well as sensory and neurological systems degradation. Reduced cardiac output and decline in skeletal muscle oxidative capacity are implicated in the VO_2_ max decrease observed in older adults [5]. Decline in muscular mass and consequently in strength, especially in the lower limbs, can impair mobility and postural control of seniors [6]. Age-related alterations also concern sensory systems involved in the detection of body displacements, resulting in an impairment of sensory information integration that might cause dizziness [7,8] and increase postural imbalance and the risk of fall [9].

The vestibular system is the primary sensory system involved in the balance control. Balance is maintained by implementing some basic reflexes, namely Vestibulo-Spinal Reflexes (VSR) dedicated to postural stabilization, and Vestibulo-Ocular Reflexes (VOR) involved in ocular compensation for movement, which allows for unblurred vision during motion [10,11]. Vision and proprioception collaborate with the vestibular system in order to maintain balance during locomotion and everyday activities. Due to multiple physiological, anatomical and behavioral reasons, all of these systems lessen in efficiency with advanced age [12,13,14].

Physical inactivity and sedentary lifestyle are associated with many health risks, increasing the possibility of cardiovascular and metabolic diseases, cancers, and negatively affecting bone and muscle structure as well as psychological health [15,16]. Inversely, exercising and physical activity have multisystem anti-aging effects during the aging process, attenuating many of its deleterious systemic and cellular effects, and preventing or hampering the development of many age-related diseases [17].

Adapted physical exercise programs, including resistance, balance, and flexibility exercises, have demonstrated benefits for functional capacities of the senior population [18,19,20]. Physical exercise has also been shown to significantly improve static and dynamic balance in older participants (>65 years old.) [21,22]. Both long and short programs revealed improvement in postural control [21,23]. Gauchard et al. [24] examined the impact of regular physical activity on vestibular information usage and their relation to postural control in older adults. VOR and postural stabilization were evaluated in healthy senior subjects who regularly practiced low-energy (yoga, soft gymnastics) or bioenergetic (swimming, cycling, running) physical activities compared to the control group. Their results demonstrated better postural control with higher vestibular sensitivity, and less visual dominance in sensory strategies in subjects practicing regular physical activities, with the vestibular sensitivity and visual independence being especially important in the low-energy exercise group [24]. Therefore, physical activity helps older adults gain muscle strength and flexibility, as well as better vestibular sensitivity and greater vestibular and less visual impact on postural control. These functional improvements lead to amelioration of balance and reduction of risk of fall, and counter the loss of autonomy.

Despite all the benefits of regular physical activity and exercise, classical face-to-face training is not always accessible for older adults. Living in rural area can may be a factor complicating the access to training centers [25]. The cost of training can be a limiting factor by itself for the most disadvantaged seniors [26]. Reduced autonomy can also prevent seniors from moving to appropriate training centers [26]. In addition, the traditional exercise and training environment is not always adapted to the physical condition of the seniors and can lead to injuries and other harm [27]. The current COVID-19 pandemic restrains the possibility of outside or collective physical exercise, leading to even greater sedentarization of older adults [28,29,30]. Remote supervised physical exercise training thus could become an optimal solution.

Physical exercise programs delivered by Videoconferencing have been used with patients or post-operative populations, but rarely with seniors for loss of autonomy prevention [31]. A study by Wu et al. [32] compared the effectiveness of exercise programs: Face-to-Face, by Videoconference, or using videotapes. The subjects of this study underwent Tai-Chi 15-week exercise programs delivered Face-to-Face with a group and a trainer, by Videoconference in real-time with a group and a trainer, or autonomously at home using videotape recordings of the training. Both Videoconference and Face-to-Face modes significantly improved physical condition and self-perceived health of subjects, as well as reduced the mean number of falls post-training. The unsupervised videotapes-based mode was shown to be significantly less effective and led to weak adherence [32]. We could conclude that videoconferencing exercise programs, providing real time online support and social interaction in the participant’s home, can be an optimal solution for seniors for whom the classical face-to-face mode is not suitable, representing an accessible and effective way to improve health and performance. However, unlike exercising with a trainer Face-to-Face, the screen-based format of training implies fixing the gaze at a moving image on a screen during active self-motion. It has been clearly demonstrated that recurrent simultaneous visual and vestibular stimulations, as well as visual suppression of the VOR, can temporarily alter the mechanism of sensory information processing [33,34,35,36,37]. Therefore, it is possible that the screen-based videoconference training can induce a different effect on visual-vestibular processing than the classical Face-to-Face format. The change of sensory processing strategies might affect postural control efficiency, and therefore become a danger to older adults during, and for a short time after, training with the use of videoconferencing.

Therefore, the first objective of this study was to compare the effectiveness of exercise programs delivered Face-to-Face and by Videoconferencing on functional capacities and balance in the senior population. The second objective was to evaluate if the Videoconference-based mode affected the processing of sensory information that could affect postural control. The hypotheses of the study were that the Videoconference-based mode of training would improve functional capacities and balance in senior subjects as well as the Face-to-Face mode, and that the vestibular system characteristics would be modified in the Videoconference group and not in the Face-to-Face group.

## 2. Materials and Methods

### 2.1. Participants

Thirty voluntary participants aged between 66 to 79 years old were recruited. All participants presented no vestibular, neurological, oculomotor, and uncorrected visual and/or auditory pathologies. Mental illness, recent hospitalization, and regular physical exercising (>1.5 h per week) were also criteria of exclusion. During a medical examination, the study procedure was explained in detail, and written consent was obtained from all participants. The protocol was approved by the National Ethics Committee for the Research in Sciences and Techniques of Physical Activity and Sports (CERSTAPS, #2016-19-04-12). Subjects were randomly divided into two groups: a Face-to-Face Training group (FT) (*n* = 15, 72, adherence rate 100%, 70 ± 3.56 years old; 70.68 ± 15.21 kg; 165.47 ± 8.85 cm), and a Videoconference Training group (VT) (*n* = 13 (2 dropped out during the protocol; adherence rate 86.7%), 73.23 ± 4.00 years old; 75.88 ± 10.92 kg; 163.00 ± 8.66 cm). The FT group followed an exercise program Face-to-Face with a trainer at the participant’s home, while the VT group used an equivalent training program by Videoconference developed by specialists in remote Adapted Physical Activity (Siel Bleu-Motion^®®^, Colombelles, France).

### 2.2. Study Design

This was a before–after design study. After the inclusion and random group attribution, all participants were tested in laboratory conditions before (T1) the training. Peak oxygen uptake, maximum workload, maximum heart rate reached, isokinetic strength of the knee flexor and extensor muscles, isometric strength of ankle flexion and extension, vestibulo-ocular reflex, and postural control under static and dynamic conditions were evaluated. The training program followed basal evaluation. After accomplishing the training program, the subjects were tested in laboratory conditions again (T2).

The Videoconference system for the videoconference group was provided, installed and configured by the research team at each participant’s home before the study. The system was easy to use for the participant non-familiar with the technology, having only a “GO” button to push to launch the videoconference at the training time. The system was developed by the European MOTION project—Remote Home Physical Training for Seniors. Specialists in physical activity were provided by the associative group Siel Bleu. The assessments of the physical condition were performed by researchers specialized in physical activity and the evaluations of the peak oxygen uptake were effectuated by a sports and exercise physician. The training material such as ergo-cycles and steppers for the aerobic part of the training were also provided by the research team. The exercise intensity in the videoconference mode was monitored with the help of the web-camera and the HR monitor by a coach during the training.

### 2.3. Physical Condition Assessment

#### 2.3.1. VO_2_ Peak Assessment

Peak Oxygen uptake (VO_2_ peak) was evaluated with a test on a bicycle ergometer (Ergoline er9000^®®^, Ergoline GmbH, Bitz, Germany) performed under medical supervision. The test was triangular and continuous with direct measurement of maximal levels of respiratory gas exchange. Participants sat on the bicycle ergometer and were instructed to pedal as long as possible with gradually growing resistance settings. The ergometer resistance was initially set to 30 Watts and increased by 20 Watts every 2 min. The resistance levels were recorded throughout the task, and the maximum was retained.

Oxygen and carbon dioxide flows were measured with a respiratory mask. The measurements were taken continuously before the test (baseline measurements), throughout the test (maximal values) and for 3 min of passive recovery on the bicycle after the test (recovery measurements). The heart rate and electrocardiogram were recorded continuously to track possible cardiac irregularities and stop the protocol, if necessary.

Achieving plateau on heart rate and VO_2_ recordings, or inability to follow the imposed pedaling velocity or a given resistance served as criteria for reaching the VO_2_ peak [38]. The major criteria for reaching the VO_2_ peak was when the increase in oxygen consumption accompanying the increase in the ergometer resistance did not exceed 100 mL/min and the respiratory quotient was above one [39].

The maximum oxygen uptake (VO_2_ peak, mL/min/kg) measured during the test was the major parameter of evaluation of the aerobic capacity of a subject.

#### 2.3.2. Muscular Assessment

The isokinetic strength of the knee flexors and extensors was evaluated with a dynamometer Cybex NORM. For the isokinetic strength of the knee flexors and extensors evaluation, maximal torque (N m) was measured. Subjects sat on an ergometer with the right leg fixed. The participants were instructed to make maximal concentric knee flexions and extensions at 60°/s (3 repetitions) and 240°/s (5 repetitions). The two trials at 60°/s and 240°/s were separated by 60 s break. The best result was retained.

Ankle plantar and dorsal flexors Maximal Voluntary isometric Contraction (MVC) was evaluated with an ergometer built in the laboratory for this purpose. The ergometer was calibrated using calibrating masses of known weights (More details about the ergometer are provided elsewhere [40,41]). For the plantar and dorsal ankle flexors evaluation, maximal voluntary isometric contraction (MVC, N m) was measured. Subjects sat on an ergometer in the standard isometric position (90° between the leg and foot and the knee bent at 5 to 15°, 0° for the leg in complete extension). One limb was evaluated at a time. The participants were instructed to push a pedal of the ergometer (“like an accelerator pedal”) as strongly as possible for 5 s, then to pull as strongly as possible for 5 s. A pause of 30 s was allowed between the trials, and three trials were made for each ankle. The best result was retained.

### 2.4. Vestibular Assessment

Vestibular function can be evaluated with the VOR recordings in response to head rotations. These eye movements in the direction opposite to the direction of rotation of the head are called the slow phases and are followed by rapid phases, re-centering the eye position. Succession of slow and rapid phases forms a vestibular nystagmus. The Time Constant (TC) of the VOR decay and the gain of the nystagmus reflect the efficiency of the ocular compensation.

The rotatory test with a step-stimulation protocol was used to stimulate the vestibular system using the rotatory chair Giga Torque. Measurements of eye nystagmic movements were performed with the video-nystagmography system NysStar1 (Instrumentation DIFRA, Eupen, Belgium). The observations were held in complete darkness to avoid any visual stimulation.

Participants sat on the rotatory chair secured with a harness and wore the video-nystagmography goggles. The chair rotation followed a trapezoidal step-stimulation protocol that consisted of two phases (in clockwise and counter-clockwise directions) of rotation of 90 s, with 90 s pauses after each deceleration to allow the post-rotatory nystagmus to decay. The plateau velocity of the chair was 60°/s, and the acceleration and deceleration were at 100°/s^2^. The order of clockwise and counter-clockwise stimulation was counterbalanced to avoid the effect of the order.

Calculation of the compensatory nystagmus velocity was performed with the differential filter (step = 50 ms). After that, slow phases of nystagmus were detected automatically on the eye-movement tracks with the help of PRO-Line DiSoft II software (Instrumentation DIFRA, Eupen, Belgium) with consequent manual verification and correction.

**Figure 1:**
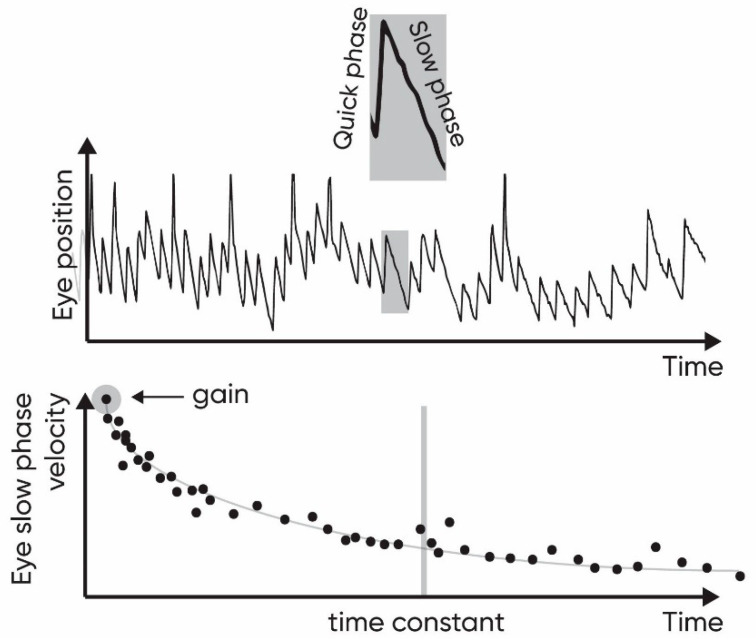
The ocular movement track of position (**upper** panel) and slow phase velocity (**lower** panel). The two parameters evaluated are the gain and the time constant of the slow phase velocity of ocular compensation.

The parameters to estimate the efficiency of ocular compensation for the movement were the time constant and the gain of the ocular compensation. Both parameters were estimated for the right semicircular canal by averaging the clockwise per-rotatory nystagmus parameters and counter-clockwise post-rotatory nystagmus parameters, and inversely for the left semicircular canal. The curve of the slow phase velocity plotted as a function of time decreases exponentially (Figure 1, lower part). The TC is estimated as the time at which the area subtended by the slow phase curve is 63% of the total area subtended by the entire curve [42]. The gain is the ratio of the initial maximal slow-phase velocity of eye movements appearing at the onset of the acceleration or deceleration to the head velocity. The maximal velocity was marked manually and all the previous nystagmus were excluded. The slow phases are identified until the complete decay or inversion of the nystagmus.

### 2.5. Postural Control Assessment

Posturography is a postural stability assessment technique, which can use static or dynamic conditions to qualify the balance. A force platform is usually used as a tool to assess postural stability. In this study, we used the SPS Synapsys platform (Synapsys, Marseille, France) to perform the measurements.

Participants stood straight on the platform with their feet apart at an angle of 30° and kept their arms at the sides while looking straight ahead. In this position, the participants had to maintain their balance under four different conditions. At each condition the stability was measured with eyes open and then with eyes closed.

With the static platform protocol, subjects were instructed to stand on the static force platform for 20 s.

With the unstable platform protocol, subjects were instructed to stand on the force platform that is unstable in Medial-Lateral (ML) axis or Anterior-Posterior (AP) axis for 51 s.

With the dynamic “trapezoidal” velocity platform protocol, subjects were instructed to stand on the force platform moving along the Anterior-Posterior axis for 51 s. During 51 s, the force platform effectuated 6 AP translations. The anterior and posterior translations were alternated, each translation resetting to the center and taking 8 s. The velocity of the platform displacement was 0.07 m s^−1^.

With the dynamic “sinusoidal” velocity platform protocol, subjects were instructed to stand on the force platform moving along the Anterior-Posterior axis for 51 s. The platform oscillated along the Anterior-Posterior axis continuously with the frequency of 0.25 Hz.

The sway area (SA, mm^2^), and the Standard Deviation (SD, mm) of the sway of the center of pressure were analyzed under Static condition along Medial-Lateral (ML) and Anterior-Posterior (AP) axes, and along the axis of the platform movement (AP) under Trapezoidal and Sinusoidal conditions. The Standard Deviation (SD, °) of the platform tilt was evaluated for the unstable condition along ML and AP axes. In order to evaluate the role of visual information in maintaining the static posture, the quotient of Romberg (RQ) was calculated as a ratio between the surface parameter of the center of pressure with EC to the one measured with the eyes open EO during the static condition.

### 2.6. Training

FT and VT groups underwent the training program for sixteen weeks. The program proposed one-hour training sessions twice a week. Training took place at the participant’s home either face-to-face with a trainer for the Face-to-Face group, or by Videoconference with a group of 4 participants and one coach, between 2 p.m. and 5 p.m. on the same day of the week and the same hour during the whole protocol.

The training program was progressive, introducing new variants of exercise and demanding interaction with coach and other participants throughout the exercise sessions. The training was based principally on aerobic and resistance exercises with the inclusion of postural balance exercises. The aerobic part of the training included exercises with ergo-cycles and steppers. The ergo-cycles included a HR tracker that allowed monitoring of the intensity of exercises and ensure participants stayed within the 60–85% range of the maximum heart rate. The resistance exercises were composed of variations of five types of exercise including sitting-standing transitions, arm resistance workout with elastic bands, squats, lunges and plank exercises. Balance exercises consisted of variations of eight types of exercise including lying-sitting-standing posture transitions, jumps, high-stepping, object picking and others, and included exercises with eyes closed. As the exercises were composed of multiple variants, the intensity was increased by adding complications, such as performing with eyes closed or with one foot in the air.

In more detail, the physical exercise program and the technological development for the project is described elsewhere [43].

### 2.7. Statistics

We analyzed the evolution of the VO_2_ peak, muscular strength, VOR and posturography variables with repeated ANOVA measures with group (FT, VT) as a between-subject factor and pre/post training measurements (T1, T2) as a within-subject factor for 28 subjects (26 for the VOR evaluations and 27 for the knee flexors and extensors evaluation, due to data recording or organization issues). The post-hoc test with Holm correction was used if a significant factors interaction was observed. The values are presented as Means ± SD. The threshold of statistical significance was set at *p* < 0.05. All statistical analysis was performed with JASP (version 0.11.1, JASP Team, Amsterdam, The Netherlands).

## 3. Results

### 3.1. Physical Condition

The mean and standard deviation values for the physical condition evaluations are presented in the Table 1.

VO_2_ peak was significantly increased by training (F(1, 25) = 4.37; *p* = 0.05), with no effect of group or the interaction of factors.

Maximum workload was also significantly increased by training (F(1, 25) = 8.48; *p* = 0.007), with no effect of group or the interaction of factors.

No differences in the maximum heart rate reached were found.

Maximal torque of the knee flexors at 60°/s (F(1, 26) = 5.04; *p* = 0.03) was significantly increased by training, with no effect of group or the interaction of factors. Knee extensors torque at 60°/s does not seem to be significantly modified. Knee flexors (F(1, 25) = 12.02; *p* = 0.002, Table 1) and extensors (F(1, 25) = 13.00; *p* = 0.001) at 240°/s were both significantly increased by training, with no effect of group or the interaction of factors. The ankle plantar flexors also showed a significant increase in MVC (F(1, 26) = 21.70; *p* < 0.001) between T1 and T2, with no effect of group or the interaction of factors. The ankle dorsal flexors’ strength was not modified.

### 3.2. Vestibulo-Ocular Reflex

The mean and standard deviation values for the VOR evaluations are presented in Table 2.

The analysis of variance showed a significant decrease (F(1, 24) = 5.06; *p* = 0.03) of VOR time constant for the left semicircular canal between T1 and T2, with no effect of group or the interaction of factors. No modifications were observed for the time constant of the right semicircular canal, nor for the gains of the ocular response (Table 2).

### 3.3. Posturography

The mean and standard deviation values for the posturography are presented in Table 3.

The analysis of variance has not shown any change in the SA, SD on the static platform, and the Romberg Quotient both with eyes open and with eyes closed, along both AP and ML axes.

For the unstable force platform, the SD along the AP axis with eyes open has shown a marginally significant effect of training (F(1, 26) = 4.17; *p* = 0.05) and a significant interaction of training and group effects (F(1, 26) = 7.40; *p* = 0.01). The post-hoc analysis revealed a significant decrease of SD by training (t = 3.25, Holm’s corrected *p* = 0.019) for the VT group. No modifications in the SD along AP axis with eyes closed were detected. The SD along ML axis with eyes closed was significantly decreased by training (F(1, 26) = 6.43; *p* = 0.02) with no effect of group or the interaction of factors. No modifications in the SD along ML with eyes open were detected.

For the dynamic platform with trapezoidal protocol, the analysis of variance has shown a significant training and group effects for the SD along the AP axis with eyes open (F(1, 26) = 4.54; *p* = 0.04) and eyes closed (F(1, 26) = 4.65; *p* = 0.04), while main effects were not significant. The post-hoc analysis demonstrated a significant decrease in SD (t = 2.87, Holm’s corrected *p* = 0.05) for the FT group only, with eyes open. No modification of the postural sway area was detected.

For the dynamic platform with sinusoidal protocol, the sway area with eyes open was significantly decreased by training in both groups (F(1, 26) = 4.99; *p* = 0.03) with no effect of group or the interaction of factors. The sway area with eyes closed and SD with eyes closed or open were not modified.

## 4. Discussion

The present study aimed to compare the impact of physical exercise training delivered Face-to-Face and by Videoconference on functional capacities and balance and to evaluate possible effects of the Videoconference-based mode on the processing of sensory information that could affect postural control in the senior population.

We observed a significant increase in the functional capacities of the participants after the training, based on the VO_2_ peak evaluations, as well as maximal and isokinetic strength of lower limbs in both groups. Surprisingly, the time constant of the left semicircular canal VOR was slightly diminished in both groups. We also found a significant diminution of the postural sway area with the sinusoidal platform acceleration protocol with eyes open, and of the maximal amplitude and SD of the unstable force platform tilt with eyes closed, which can indicate a slight amelioration of the postural balance equal for both groups. At the same time, some parameters were modified differently by the two protocols. The SD with eyes open in AP axis with the unstable platform has diminished more significantly in the Videoconference Training group, while the SD with eyes both open and closed along the AP axis with the trapezoidal protocol have diminished further in the Face-to-Face Training group. These differential modifications for the two groups might result from the differences in two training modes, such as the differences in social involvement and the configuration of the training environment.

To address the hypothesis that both Face-to-Face and Videoconference-based mode can improve functional capacities and balance, we evaluated the maximum oxygen uptake, lower limbs strength, and postural stability in all participants before and after the training period. The training improved the VO_2_ peak in both groups, similarly to the effect reported previously for combined training [44]. VO_2_ peak diminishes with age which leads to difficulties in performing everyday activities when it drops below 15 mL kg^−1^ · min^−1^ [45]. The population in the present study had VO_2_ peak far above this critical level, so the training program allowed the participants to gain an additional distance from deconditioning thresholds. The training also improved the neuromuscular performance in lower limbs. Isokinetic knee torque of knee flexors at 60°/s and both flexors and extensors at 240°/s were improved in both groups with training and the values meet observations found in the literature [46,47]. Knowing that both modes contained similar training protocol, it can be concluded that both Face-to-Face and Videoconference training courses can be effective in improving the aerobic capacities and muscular strength in the senior population. This conclusion agrees with several previous findings, indicating that supervised home-based training is effective and can slow down the loss of autonomy in older adults [32,43]. Training was also found beneficial for maintaining postural balance in dynamic conditions. Postural sway area in the sinusoidal platform acceleration protocol with eyes open, as well as the maximal amplitude and SD of the unstable force platform tilt with eyes closed, were significantly diminished after the training for both groups. This amelioration demonstrates that even if the protocol was not specifically dedicated to balance performance, the subjects participating in the study have improved some of the aspects of postural control. The increase in muscular strength in the knees and ankles, largely involved in maintaining the posture in seniors that underwent both training protocols, could partially explain the amelioration of postural control in dynamic conditions [40]. At the same time, some parameters were modified differently by the two protocols. The SD with eyes open in AP axis with the unstable platform has diminished more significantly in the Videoconference Training group, while the SD with both eyes open and eyes closed along the AP axis with the trapezoidal protocol have diminished further in the Face-to-Face Training group. It is possible that the difference in the mode of training could induce these differences. The FT group showed a greater stability during the trapezoidal dynamic platform stimulation, which includes sudden destabilizing accelerations. Indeed, exercising with the physical support of a trainer can lead to taking greater risks at exercises challenging postural control with less fear of falling. This can indeed result in better training of the postural system in dynamic and perturbing postural situations. Otherwise, this can result from a less adapted training environment at home and more restricted space, because participants needed to stay in the visual field of the web-camera. Therefore, the Face-to-Face group could develop better postural balance skills and a better reaction to the sudden passive stimulations than the VT group due to the reassuring physical presence of the coach during training. Interestingly, however, the VT group has shown better balance control with the unstable platform than the FT group, so the training through videoconference was beneficial for postural control.

Besides the benefits brought by APA, exercising using the videoconference needs to be safe. The physical training using a videoconference system implies head movements together with the visual fixation on the screen of a computer, where the images do not necessarily move in the same direction or at the same speed as the participants. Knowing that repetitive simultaneous visual and vestibular stimulations, as well as visual suppression of the VOR, can potentially provoke rearrangement in visual-vestibular processing [33,34,36,37], which can be an important factor in the weakened postural control, it was suggested that the Videoconference Training group could develop modifications in the vestibulo-ocular responses.

To address the hypothesis that the use of Videoconference for supervised exercising might induce some particular sensory reorganization, different from the classical Face-to-Face mode, we evaluated the VOR gains and time constants and differences in postural stability in subjects from both groups before and after the training period. The Romberg quotient that shows the relative importance of the visual component for maintaining the postural balance was calculated from static postural data. The Romberg quotient is an important parameter, especially in the older adult population, as it tends to increase with age, as postural control becomes more and more visually-dependent, which can, however, be counteracted by regular physical activity and exercise [24]. It has been shown that the Romberg quotient can serve as a reliable index of distinction between faller and non-faller population [48]. Literature shows that the Romberg Quotient might be modified with a dedicated training program in 12 weeks [49]. In our study, no modifications of the Romberg quotient within either group were detected in subjects, which indicates that sensory integration for postural control was not altered. It has been shown that seniors present alterations in vestibular function in comparison to younger subjects [50]. Aging is characterized by a less efficient vestibulo-ocular and optokinetic reflexes and a decreased ability of visual suppression of VOR. More specifically, the study by Baloh et al. [50] showed that the ocular response during a rotary vestibular stimulation decreased with aging from a TC of 15.1 s and a gain of 0.63 for young subjects (mean age 26 ± 6.7 years old) to TC of 11.7 s with a gain of 0.58 for the elderly (mean age 79.5 ± 4.3 years old). These changes can have functional consequences such as impaired balance or vertigo. In the current study, the average TC for all groups combined was about 14 s with a gain of 0.71. These parameters are therefore slightly higher than those observed in the study of Baloh et al. [50]. However, the average age of our participants was 72 ± 3.75 years old. The difference in age between the populations of these two studies could partly explain the differences in TC, especially since it has been shown that the degradation of the vestibular system accelerated from the age of 80 [13]. Therefore, our data is consistent with previous observations. In addition, the participants recruited in the current study were in good general health that could also explain the differences in TC observed. The VOR evaluations after the training have surprisingly shown a slight unilateral diminution in the VOR time constant for the left semicircular canal in subjects, and this modification was detected regardless of the training mode. This indicates that the modification might be provoked by the training itself, but not the mode of the training delivery. The training protocol for both groups included 180° rotation jump. In order to avoid falls or loss of balance, the direction of jumps with rotations was not imposed so that participants could choose their preferential side. We suggest that the participants dominantly chose rotations to the right, which could provoke slight unilateral habituation of VOR to the left that is observed in both groups of the study. Therefore, no Videoconference-related sensory processing modifications were detected in the subjects. The Videoconference-based physical exercise training thus can be considered safe for older adults.

This study has appreciated the comparable efficacy of Face-to-Face and Videoconference-based physical training protocols for the amelioration of physical condition of the seniors, and the absence of specific sensory reorganizations for the Videoconference training mode. Therefore, supervised exercising using Videoconference can be considered a safe and effective option for seniors who cannot access the Face-to-Face training for any reason. This study has, however, some limitations. First, due to dropout of two subjects who did not continue the training, the study design was unbalanced which could affect statistical power of tests (13 vs. 15 subjects). The dropouts could probably be explained by the less involving character of the remote social interactions. The adherence rate of 86.7% is nevertheless typical for this mode of training [32,43]. Second, the mechanisms by which both training conditions can lead to different results in postural control can only be hypothesized, and future studies should address this topic. Moreover, failing to detect modifications, in this case, the sensory processing modifications detectable with the VOR and the Romberg Quotient evaluation, does not prove the actual absence of modifications. To assure the safety and evaluate the question in detail, confirmation by other studies of this type is required to assure reproducibility and to evaluate the possible effect of the Videoconference training mode on the VOR with a greater number of subjects across different physical training protocols through Videoconferencing.

The safety issue that was the focus of the study was the sensory modification by the use of Videoconference while exercising. While no videoconference-related modification was detected, a unilateral diminution of VOR time constant, which most probably reflects the unilateral habituation [34], was detected independently of a group. Thus, we would like to emphasize the importance of a thorough design of the exercises involving rotations for the physical training for older adults, as it was shown that such habituations can be provoked by as short as ten sessions and persist for no less than a month [51]. The uncontrolled or systematically unilateral choice of direction of rotations can potentially lead to the development of sensory asymmetries.

## 5. Conclusions

The Face-to-Face and the Videoconference-based adapted physical exercise training was shown to improve the general physical condition of all the subjects, by increasing VO_2_ peak and muscular strength and improving postural control in both groups. The use of the Videoconference-based training does not seem to have altered the vestibulo-optic sensory interactions in subjects, or the portion of the visual system used in postural control. Therefore, the Videoconference-based supervised exercise program can be a safe and effective way to maintain good functional capacities in seniors. The benefits of this remote type of training are clear: improving performance with a trainer and socializing with a group with no need to leave home can be a good solution when going elsewhere is problematic.

## Figures and Tables

**Table 1:** Values of the evaluation of aerobic and muscular capacities (Mean ± SD) for Face-to-Face Training Group and Videoconference Training Group before (T1) and after (T2) the training period. VO_2_ peak is the rate of oxygen consumption by kg of body. HR max—maximal heart rate reached. MVC—maximal voluntary contraction. Sign.—Significance level. * *p* ≤ 0.05, ** *p* ≤ 0.01, *** *p* ≤ 0.001; a: intervention effect (corresponds to a significant effect of intervention for both groups).

Functional Capacities:			FT Group		VT Group		Sign.
			T1	T2	T1	T2	
Aerobic capacities		VO_2_ peak, mL/kg/min	22.93 ± 7.82	23.86 ± 8.04	19.54 ± 5.21	21.46 ± 4.58	*a
		Maximum workload, Watt	110 ± 37.42	117 ± 35.67	95 ± 24.21	101 ± 23.09	**a
		HR max, bpm	153 ± 14.26	155 ± 12.07	147 ± 8.16	149 ± 16.65	
Knee	60°/s	Extension, N m	101.93 ± 41.81	111.27 ± 37.38	92.92 ± 24.11	92.62 ± 22.39	
	60°/s	Flexion, N m	55.07 ± 21.31	60.27 ± 18.77	53.39 ± 17.66	54.62 ± 18.24	*a
	240°/s	Extension, N m	53.00 ± 23.87	57.00 ± 22.08	43.85 ± 16.03	48.39 ± 13.88	***a
	240°/s	Flexion, N m	33.07 ± 14.75	35.65 ± 14.21	27.54 ± 10.47	31.23 ± 10.13	**a
Ankle (MVC)		Plantar flexors, N m	81.07 ± 30.12	94.53 ± 35.57	67.92 ± 33.59	80.73 ± 25.04	***a
		Dorsal flexors, N m	47.07 ± 18.39	46.31 ± 18.61	43.30 ± 19.12	40.27 ± 18.14	

**Table 2:** Values of the VOR evaluation parameters for Face-to-Face Training Group and Videoconference Training Group before (T1) and after (T2) the training period. TC—time constant of the ocular reflex, gain—the relation of the maximal ocular velocity to the rotatory velocity of the head, both parameters calculated for the Left and Right semicircular canals. Sign.—Significance level, * *p* ≤ 0.05; a: intervention effect (corresponds to a significant effect of intervention for both groups).

Vestibular Evaluation:		FT Group		VT Group		Sign.
		T1	T2	T1	T2	
VOR	TC Right, s	14.99 ± 4.89	14.40 ± 4.97	12.86 ± 2.37	13.33 ± 3.87	
	TC Left, s	15.47 ± 3.69	14.25 ± 3.77	14.33 ± 3.07	12.64 ± 3.30	*a
	Gain Right	0.73 ± 0.23	0.75 ± 0.11	0.72 ± 0.14	0.70 ± 0.13	
	Gain Left	0.71 ± 0.22	0.71 ± 0.17	0.69 ± 0.14	0.70 ± 0.15	

**Table 3:** Values of the postural evaluation parameters with static and dynamic posturography: unstable platform, sinusoidal or trapezoidal platform oscillations, for Face-to-Face Training Group and Videoconference Training Group before (T1) and after (T2) the training period. SA—the sway area, SD—Standard Deviation of the postural sway along the Anterior-Posterior axis (AP) and Medial-Lateral axis (ML) or of the angle of the platform tilt. EO—eyes open, EC—eyes closed. Sign.—Significance level * *p* ≤ 0.05; a: intervention effect (corresponds to a significant effect of intervention for both groups), b: interaction effect (corresponds to a significant effect of intervention that is different between groups).

Posturography			FT Group		VT Group		Sign.
			T1	T2	T1	T2	
Unstable (AP) platform	EO	SD, °	1.18 ± 0.34	1.24 ± 0.42	1.63 ± 0.56	1.22 ± 0.33	*b
	EC	SD, °	2.25 ± 0.83	2.04 ± 0.49	2.22 ± 0.47	2.19 ± 0.48	
Unstable (ML) platform	EO	SD, °	1.18 ± 0.32	1.18 ± 0.27	1.47 ± 0.39	1.30 ± 0.27	
	EC	SD, °	2.41 ± 0.77	2.046 ± 0.48	2.59 ± 0.69	2.14 ± 0.61	*a
Dynamic platform (Trapezoidal protocol)	EO	SA, mm^2^	1714.02 ± 653.73	1462.26 ± 351.57	1529.58 ± 507.21	1623.24 ± 1071.94	
		SD, mm	17.27 ± 3.56	15.01 ± 1.46	14.79 ± 2.16	15.00 ± 2.85	*b
	EC	SA, mm^2^	2221.53 ± 962.86	1825.09 ± 1108.31	1779.89 ± 673.60	1795.85 ± 808.19	
		SD, mm	17.76 ± 4.10	15.37 ± 1.93	15.47 ± 2.59	16.13 ± 3.13	*b
Dynamic platform (Sinusoidal protocol)	EO	SA, mm^2^	1834.04 ± 726.56	1532.04 ± 437.38	2219.35 ± 896.38	1964.58 ± 719.66	*a
		SD, mm	18.08 ± 3.83	16.55 ± 2.85	18.80 ± 2.97	18.16 ± 3.68	
	EC	SA, mm^2^	3431.55 ± 1474.67	3031.78 ± 1177.90	4014.30 ± 2820.79	4261.87 ± 3774.00	
		SD, mm	26.00 ± 5.74	25.25 ± 5.87	25.95 ± 5.74	26.00 ± 6.23	
Static platform	EO	SA, mm^2^	249.71 ± 158.94	212.43 ± 89.46	303.17 ± 194.46	305.90 ± 224.50	
		SD ML, mm	3.63 ± 1.35	3.57 ± 0.89	3.81 ± 1.30	3.97 ± 1.80	
		SD AP, mm	4.38 ± 1.36	3.96 ± 0.91	5.06 ± 1.90	4.92 ± 1.48	
	EC	SA, mm^2^	291.57 ± 153.18	250.90 ± 97.46	368.26 ± 257.01	330.59 ± 242.65	
		SD ML, mm	3.81 ± 1.39	3.57 ± 0.79	4.52 ± 1.76	4.18 ± 1.82	
		SD AP, mm	4.98 ± 1.25	4.69 ± 1.16	5.14 ± 1.79	4.96 ± 1.56	
Romberg quotient			1.32 ± 0.64	1.30 ± 0.64	1.30 ± 0.58	1.10 ± 0.36	

## Data Availability

The data are available at https://www.researchgate.net/publication/352901620_Videoconference-based_adapted_physical_exercise_training_is_a_good_and_safe_option_for_seniors (accessed on 25 July 2021).
